# The first flea with fully distended abdomen from the Early Cretaceous of China

**DOI:** 10.1186/s12862-014-0168-1

**Published:** 2014-08-27

**Authors:** Taiping Gao, Chungkun Shih, Alexandr P Rasnitsyn, Xing Xu, Shuo Wang, Dong Ren

**Affiliations:** College of Life Sciences, Capital Normal University, 105 Xisanhuanbeilu, Beijing, 100048 China; Palaeontological Institute, Russian Academy of Sciences, Moscow, 117997 Russia; Natural History Museum, Cromwell Road, London, SW7 5BD UK; Institute of Vertebrate Paleontology and Paleoanthropology, Key Laboratory of Evolutionary Systematics of Vertebrates, Chinese Academy of Sciences, 142 Xiwai Street, Beijing, 100044 China; Graduate University of Chinese Academy of Sciences, 19 Yuquan Road, Beijing, 100049 China

**Keywords:** Mesozoic, Blood sucking, Siphonaptera, Pseudopulicidae, *Pseudopulex*

## Abstract

**Background:**

Fleas, the most notorious insect ectoparasites of human, dogs, cats, birds, etc., have recently been traced to its basal and primitive ancestors during the Middle Jurassic. Compared with extant fleas, these large basal fleas have many different features. Although several fossil species with transitional morphologies filled the evolutionary blank, the early evolution of these ectoparasites is still poorly known.

**Results:**

Here we report a new flea with transitional characters, *Pseudopulex tanlan* sp. nov., assigned to Pseudopulicidae, from the Lower Cretaceous Yixian Formation of Liaoning Province, China. Different from the previously described pseudopulicids, *P. tanlan* has relatively smaller body size but lacking any ctenidia on the tibiae or body, while the male with comparatively smaller and shorter genitalia. On the other hand, *P. tanlan* has some characters similar to the transitional fleas of saurophthirids, such as, a small head, short compacted antennae, small pygidium and many stiff setae covering the body.

**Conclusions:**

Even though other possibilities can not be ruled out, the female specimen with extremely distended abdomen suggests that it might have consumed its last meal before its demise. Compared with other reported female flea fossils, we calculate and estimate that *P. tanlan* sp. nov. might have consumed 0.02 milliliter (ml) of blood, which is about 15 times of the intake volume by extant fleas. These new findings further support that fleas had evolved a broad diversity by the Early Cretaceous.

**Electronic supplementary material:**

The online version of this article (doi:10.1186/s12862-014-0168-1) contains supplementary material, which is available to authorized users.

## Background

After Riek reported the first flea-like insect, *Tarwinia australis,* from the Early Cretaceous of Koonwarra, Australia in 1970 [1], Ponomarenko described a flea-like fossil, *Saurophthirus longipes,* in 1976 [2]. In the past 36 years, there were no more fossil fleas described from the Mesozoic, even though thousands of other insect taxa have been reported from around the world [3-5]. Extinct Strashilidae have been described as flea relatives [6], but recently they are re-interpreted as aberrant Diptera [7]. Since 2012, several flea fossils from the Mid-Mesozoic of northeastern China shed light on the origin and evolution of the basal and transitional fleas [8-11]. They were reported to live on diverse hosts such as coexisting feathered dinosaurs, pterosaurs, primitive birds and medium-sized mammals [8-10]. It has been proposed that fleas might have originated from an extinct clade of scorpionflies [8,12]. Mesozoic fleas have been organized in three families [11]. Pseudopulicidae, possessing large body sizes and very robust piercing-sucking mouthparts, are considered as the basal and stem group of fleas [8-10]. Monotypical Tarwiniidae differ from Pseudopulicidae and Saurophthiridae in having free maxillary palp (vs. no free palp), but similar to Pseudopulicidae in having tibial ctenidia [13]. Saurophthiridae are more similar to extant fleas than pseudopulicids in the medium body size, short piercing suctorial stylet mouthparts and partially internal male genitalia (vs. completely external in Pseudopulicidae) [10,13]. Up to now, 5 genera with 8 species have been assigned to these three flea families in the Mesozoic. They were reported to live on diverse hosts such as coexisting feathered dinosaurs, pterosaurs, primitive birds and medium-sized mammals [8-10,13].

Herein, we describe a new flea with transitional morphology from the Early Cretaceous, Yixian Formation of Liaoning Province, China. The new female flea has a fully distended abdomen, suggesting it might have consumed its last meal before its demise and subsequent fossilization. Comparing this female specimen with other known female fossil fleas, we calculate and estimate the volume of blood intake by this flea. These new findings support the notion that fleas had already a broad diversity by the Early Cretaceous.

## Methods

The specimen was examined under a Leica MZ 16.5 dissecting microscope, and the photographs were taken with a digital camera system connecting with the Leica MZ 16.5. In some photos, ethanol (95%) was put on the surface of the specimen to improve clarity and contrast of details. Line drawings were prepared with CorelDraw X6 and Adobe Photoshop CS 6.0.

We summarize all known female fleas from the Mesozoic in Additional file 1: Table S1 (see in [14]). Based on the data from the Additional file 1: Table S1, the average ratio of the width of abdomen / body length is 0.28. We estimate that the abdominal width of the female of *Pseudopulex tanlan* is about 2.6 mm (9.26 * 0.28) without swelling. When it is fully distended, the abdomen width is 4.0 mm. To estimate the volume of blood intake, we select the 3rd to 7th segments (with a length of 4.46 mm) as the expandable parts, and calculate an ellipsoid volume for the abdomen (Additional file 2: Figure S1, see in [14]). We get two different ellipsoids (Additional file 2: Figures S1A and C), one has dimensions of radii of 2.23 mm, 2 mm and 2 mm (Additional file 2: Figure S1B), the other has radii of 2.23 mm, 1.3 mm and 1.3 mm (Additional file 2: Figure S1D). We calculate the change of the volumes between these two ellipsoids based on the following formula: **V** = **4**/**3** * **π** * **a** * **b** * **c** (a, b, c represent the three radii of the ellipsoid, π = 3.14). In our ellipsoids, the volume change is: V1 = 4/3 * 3.14 * 2.23 * 2 * 2–4/3 * 3.14 * 2.23 * 1.3 * 1.3 ≈ 22mm^3^. Therefore, we estimate the volume of blood intake by *Pseudopulex tanlan* is nearly 0.02 cc (ml). (see the Additional file 1: Table S1 and Additional file 2: Figure S1, are available at https://datadryad.org, doi: 10.5061/dryad.q4jv0, see in [14]).

## Results

This published work and the nomenclatural acts it contains have been registered in Zoobank (http://zoobank.org): Publication - urn:lsid:zoobank.org:pub:C9B16650-CFCD-4712-B3FD-F7986A91C472;new species - urn:lsid:zoobank.org:act:65C13577-AB40-4102-8970-8AB321BB70CC.

### Description of the specimens

Insecta Linnaeus, 1758.

Siphonaptera Latreille, 1825.

Family Pseudopulicidae Gao, Shih & Ren, 2012.

### *Pseudopulex* Gao, Shih & Ren, 2012

*Pseudopulex tanlan* Gao, Shih, Rasnitsyn & Ren sp. nov.

#### Etymology

‘Tanlan’ means ‘avaricious’ in Mandarin Chinese, referring to the fully distended abdomen and possibly voracious feeding.

#### Diagnosis

Medium body size (about 10 mm long), head and thorax relatively small. Body covered with stiff, short bristles and setae. Tibia without ctenidia. Distal abdominal segments not sclerotized. Female with fore tibia about half as long as femur, cerci distinct, reaching end of abdomen. Male with relatively small and short genitalia. Mouthparts unknown.

#### Type material

Holotype female, No. CNU-SIP-LL2013002; paratype (allotype) male, No. CNU-SIP-LL2013003. Only a single plate exists for each specimen. They are housed in the Key Laboratory of Insect Evolution and Environmental Changes at the Capital Normal University (CNU).

#### Locality and age

Both holotype and paratype (allotype) were collected from the Early Cretaceous Yixian Formation, Dawangzhangzi Village, Lingyuan City, Liaoning Province, China, which was dated to be 124.6 ± 0.1 Myr [15,16].

#### Remarks

The new species differs from *P. jurassicus* and *P. magus* in: small body size (about 10 mm long), short body vestiture, relatively short female fore tibia and small male genitalia. It differs from *Hadropsylla* Huang et al. [7] in presence of distinct cerci and small distinct pygidium in female (vs. invisible in *Hadropsylla*). Additionally, it differentiates from *Tyrannopsylla* Huang et al. [7] in apical abdominal segments not sclerotized and in female cerci reaching abdominal apex (vs. female *Tyrannopsylla* cerci not reaching abdominal apex, segment 8 sclerotized laterally and segment 9 at least dorsally, and male sterna 6–9 sclerotized).

#### Description

**Figure 1:**
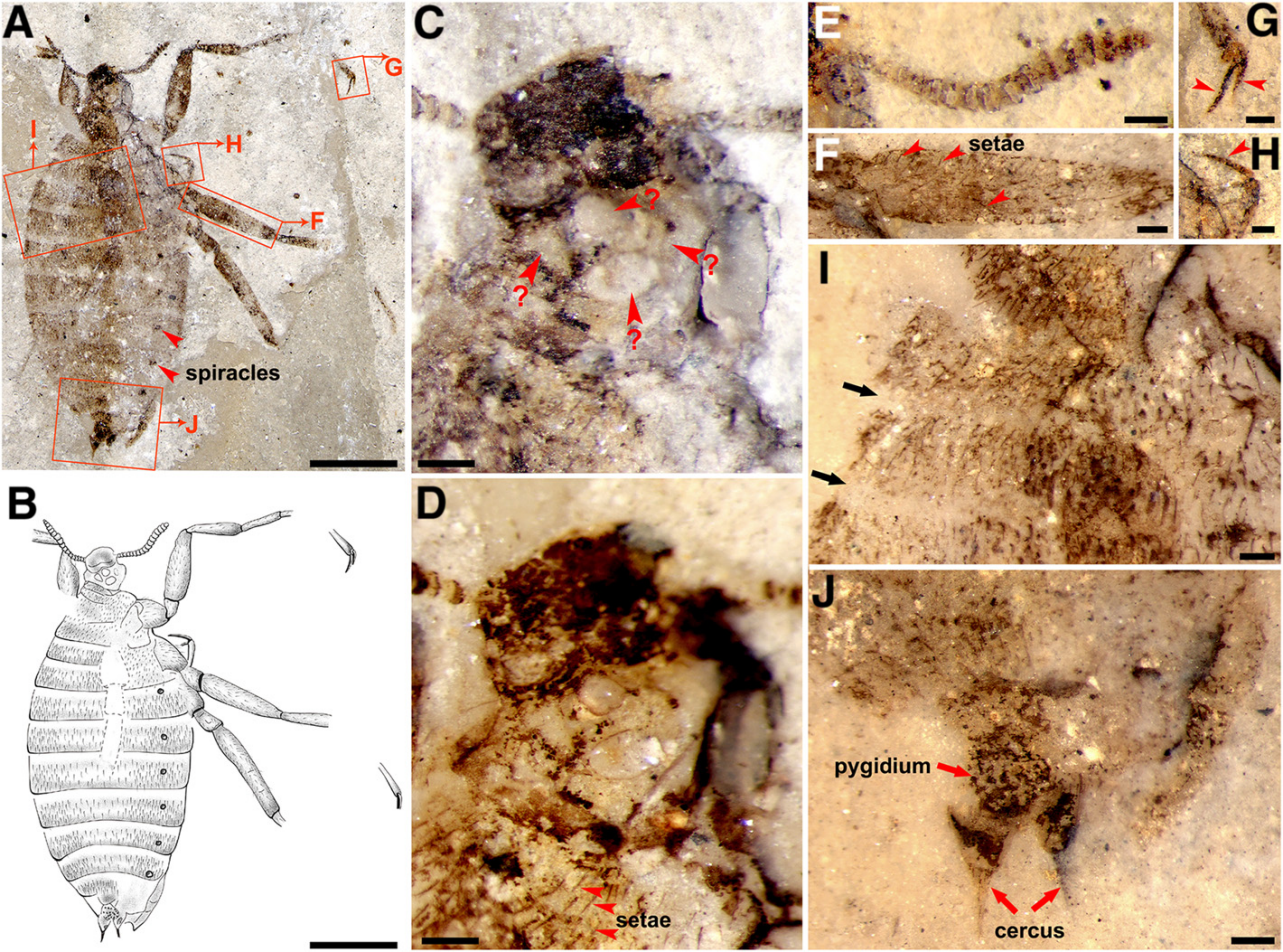
**The holotype female** ***Pseudopulex tanlan*** **sp. nov.** The specimen is from the Early Cretaceous (Yixian Fm.) of northeastern China. No. CNU-SIP-LL2013002, (**D – J**, under alcohol). **(A** **and** **B)** Photograph and line drawing; **(C** **and** **D)** Photograph of the head, **C**, without alcohol, **D**, under alcohol; **(E)** antenna; **(F)** femur of the mid-leg; **(G** **and** **H)** claw; **(I)** the segmental boundary of the abdomen; **(J)** terminalia of the abdomen. Scale bars, **A** and **B**, 2 mm, **C** – **J**, 0.2 mm.

Holotype female (Figure 1), preserved with complete body, most of the head and partial legs. Body 9.26 mm long (excluding antennae) in oblique dorsolateral view, but head in dorsal view (Figures 1A and B). Integument only slightly infuscated, except for anterior (narrow) part of head, antennal segments, claws and vestiture dark; legs and particularly pygidium and cerci somewhat more infuscated. Head about 1.15 mm wide and 1 mm high (Figures 1C and D). Compound eyes hardly visible, probably small; ocelli absent. Four unknown ovoid window-like structures preserved on lower dorsal part of head (Figures 1C and D). Antennae (1.28 mm long) with 17 antennomeres visible, basal antennomeres longer and thinner, generally widening and shortening toward to the apex, the 7^th^ to 15^th^ antennomeres compacted and cup-shaped, the last two very small (Figure 1E). Thorax slightly wider than the head, with terga simplified and similar to abdominal terga in structure, coloration, vestiture and size, except for pronotum longer and narrower. Legs incompletely preserved except for right fore leg about 7.17 mm long, with femur about 1.5 times as long as tibia, tibia thin basal, with a long seta or a few ventral setae at apex, basitarsus slightly shorter than tibia, claws almost straight (Figures 1G and H). Legs covered with moderately long setae (Figure 1F), Hind femur (2.40 mm long) thinner than middle femur (1.90 mm long), much thinner than fore femur (1.65 mm long). Abdomen with 9 segments, widest at 4^th^ segment (Figures 1A and B) with a width of 4 mm, covered with dense and stiff setae; intersegmental membrane glabrous, clear, about 0.3 - 0.5 times as long as adjacent terga or sterna (Figure 1I), Spiracles visible as dark spots on lateroterga 2–8. Pygidium small, rounded triangular, cerci closely approaching pygidium, reaching or slightly surpassing abdominal apex, each with a bundle of setae (0.34 mm long) (Figure 1J).

**Figure 2:**
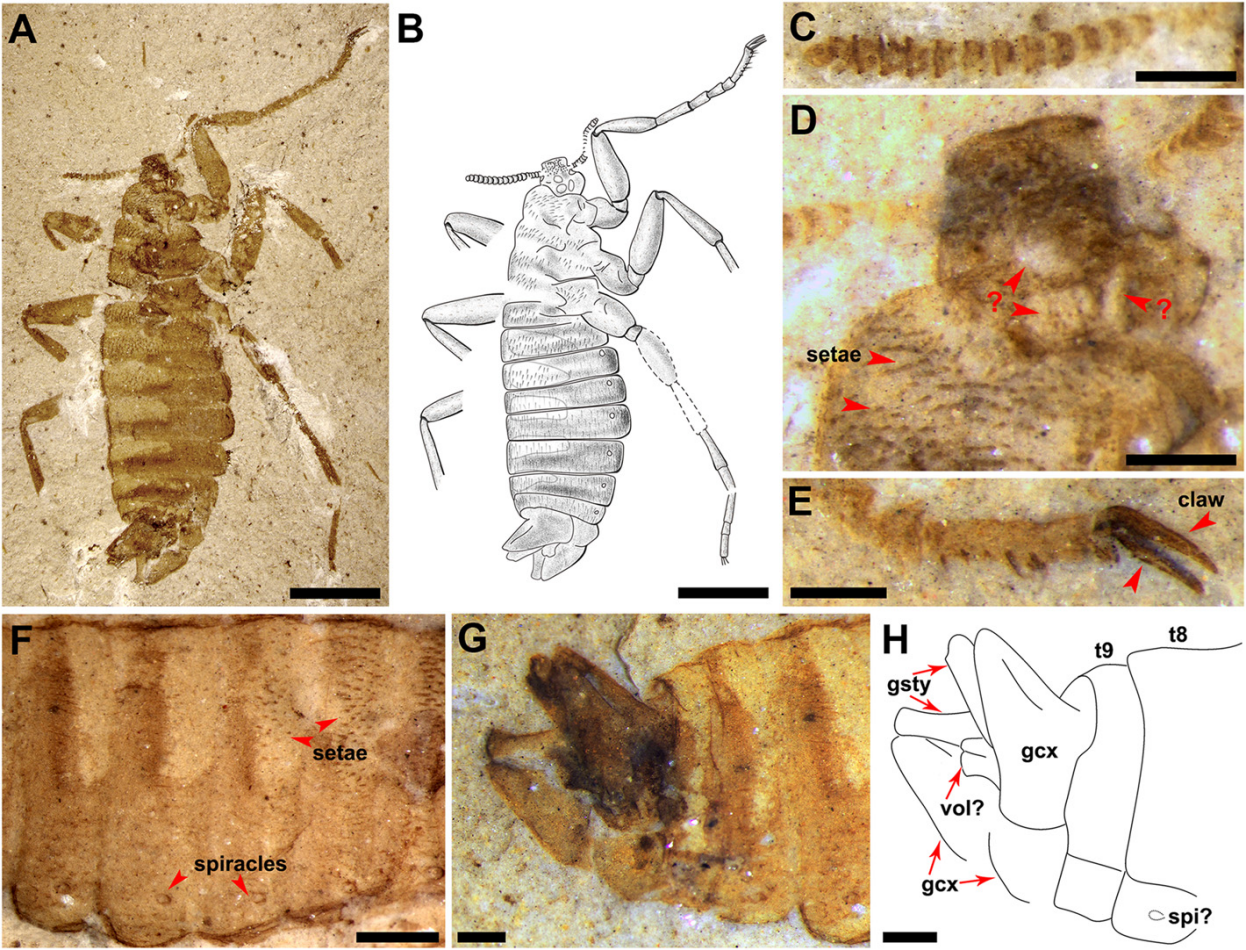
**The paratype (allotype) male** ***Pseudopulex tanlan*** **sp. nov.** The specimen is from the Early Cretaceous (Yixian Fm.) of northeastern China. No. CNU-SIP-LL2013003 (**C** – **G**, under alcohol). **(A)** Photograph; **(B)** line drawing; **(C)** antenna; **(D)** details of the head; **(E)** part of the right fore legs; **(F)** the middle part of the abdomen; **(G** **and** **H)** male genitalia. Abbreviations: gcx, gonocoxa; gsty, gonostylus; vol, volsella; spi, spiracles. Scale bars, **A** and **B**, 2 mm, **C** – **H**, 0.4 mm.

Paratype (allotype) male (Figure 2). Body about 9.5 mm long (excluding antennae) in lateral view, but prothorax and head in dorsal view (Figures 2A and B). Integuments somewhat infuscated, anterior part of head, antenna except basally, claws, vestiture and genitalia darker, but thoracic and abdominal terga paler. Antenna more uniform in width comparing with holotype female, with about 17 antennomeres (Figure 2C). Eyes not distinctly visible. The anterior part of head covered by a lot of small circular protuberance, four unknown ovoid-shaped structures apparently present on posterior part of head (Figure 2D). Thoracic structure similar to that in holotype, except that regularly thinner and longer setae not clearly visible on background of darker cuticle, making more visible stiff and short setae covering all thoracic and four basal abdominal terga (Figures 2A, B and F). Legs incompletely preserved except for right fore leg, more robust than in holotype, fore leg about 7.34 mm long, with tibia comparatively longer than in holotype. Tarsomere 5 with several (>6) stiff and sharp bristles, few setae found on other segments of legs. Lengths of femur, tibia, 1^st^, 2^nd^, 3^rd^, 4^th^, 5^th^ tarsomeres, claw of fore leg (in mm): 1.74, 1.34, 0.81, 0.54, 0.34, 0.22, 0.80, 0.50, and of the mid leg, femur and tibia 1.98 and 1.82. Abdomen 9 segmented, with spiracles located probably on lateroterga before their midlength (Figure 2F). No cercus and pygidium visible. Gonocoxa elongate triangular, with long and narrow gonostylus attached apical but visible only basally; more medial structure partially preserved probably representing volsella or penis (Figures 2G and H).

## Discussion

### Classification of *Pseudopulex tanlan* sp. nov.

Although *Pseudopulex tanlan* sp. nov. does not have ctenidia on the tibiae or body, nor preserved mouthparts, we consider it belonging to Pseudopulicidae and hence to Siphonaptera based on its wingless body covered with many stiff setae, characteristic helmet-like head, short and slightly fusiform antenna comprising compact short antennomeres, highly simplified thorax with segments similar to abdominal ones in size and form, legs with long tarsi and long, thin and gently curved claws with characteristic small basal lobe, female abdomen soft and distensible with eight well-visible spiracles and external male genitalia. It differs from other known pseudopulicids in having smaller body and head and shorter vestiture, male with shorter and smaller genitalia, and female with distinct cerci reaching the end of abdomen. Four enigmatic ovate structures are revealed at the posterior part of heads of both female and male of *P. tanlan* (Figures 1C, D and 2D). Such structures have never been observed in other insects before, and their possible function is obscure.

### Preliminary phylogeny of known fleas of Mesozoic

**Figure 3:**
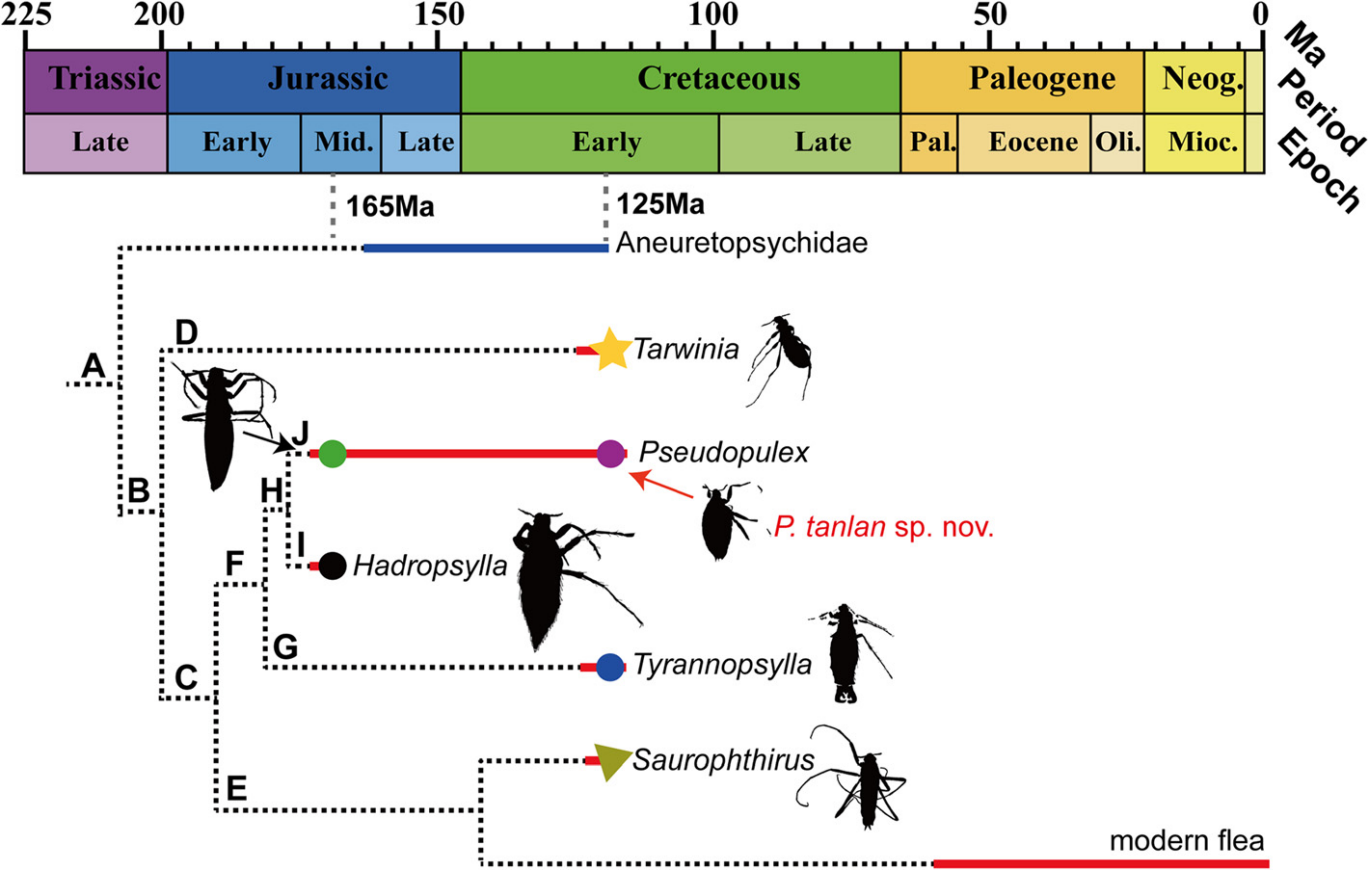
**Proposed preliminary phylogeny of the Mesozoic fleas.** Representative groups: Circle, pseudopulicids; star, tarwiniid; triangle, saurophthirids.

It has been suggested that some morphological transformations of early flea evolution are reducing their eyes and body sizes due to their ectoparasitic life style [9,10]. In the Middle Jurassic strata, all the reported fleas have large body sizes [9,11]. In the Lower Cretaceous strata, we found some fleas possessing relatively medium body sizes [1,10,11], while nearly all the modern fleas are very small, under 5 mm in body lengths [17,18]. *Pseudopulex tanlan* sp. nov. has a medium body size with length of about 9.5 mm similar to that of saurophthirids, but less than half as long as *P. magnus*. In addition, *P. tanlan* lacks ctenidia on the apical tibiae, but its dense setae on the body have a regional distribution, that is, rows of short but hard setae covering the dorsal part of thorax and the 1^st^ to 4^th^ abdominal segments, while soft and relatively long setae covering the remaining abdominal segments (Figure 2F). We consider the distinctly different distribution of setae in the new species, which are also found in saurophthirids, might be an evolutionary miniature of the common pseudocomb structures on the back of living fleas. Therefore, *P. tanlan* is supposed to be a new representative of transitional fleas within *Pseudopulex.* It is agreed that fleas evolved from a clade of winged insects [19,20]. Huang et al. proposed that the blood-sucking behavior might have evolved from feeding plant fluids [8]. Based on all described fossil fleas hitherto but constrained by insufficient and limited morphological data shown on some described specimens, a conceptual phylogeny of fleas is proposed in Figure 3 to illustrate the evolution of fleas. In Figure 3, Clade A is supported by the long proboscis with stylet mouthparts. Clade B is supported by wingless and small, simplified thorax, compact antennae, and long tarsi with long, thin, and gently curved claws bearing small basal lobe. Clade C is supported by the helmet-like head (with anterior part bearing antennae and eyes much narrowed comparing with main part of head), and labial palpi closely associated with the beak (never preserved free, unlike *Tarwinia*). Clade D (*Tarwinia*) is supported by the hard, subrectangular pronotum narrowing rearwards (less hard and narrowing forwards in Pseudopulicidae, small heart-form in males and small, weak and hardly visible in females of Saurophthiridae), short, incrassate fore and mid coxae and tibiae, very long tarsi, and ctenidia covering apical and at least distal half of the outer margin of all tibiae. Clade E is supported by the very thin and long legs with lateral position of coxae, loss of external abdominal segmentation in female, and at least partially internal male genitalia. Clade F (Pseudopulicidae) is supported by labial palpi forming mouthpart sheaths (reaching the beak apex and streamlined, standard in width, in contrast to relatively short and with well individualized segments in *Saurophthirus*) and simple band-like meso- and metanotum in both sexes (in contrast to wide heart-form, with scutellum distinctly extending backwards in male *Saurophthirus*), tarsi about as long as femur and tibia combined, female cerci short (not reaching abdominal apex) or lost, and longer body vestiture. Clade G (*Tyrannopsylla*) is supported by the less compacted antennomeres with large scape, relatively short stylets and the sclerotization abdominal sternites with greatly reduced setae. Clade H is supported by very long serrated stylets with sharp saw-blade like notches (not preserved in *P. tanlan*), short but thick coxa and the tibia shorter or equal to femur. Clade I (*Hadropsylla*) is supported by much higher number of antennomeres, extremely long stylets, shorter tarsi and tibial ctenidia not in row. Clade J (*Pseudopulex*) is supported by the abdomen with small pygidium, relatively short legs, and small male genitalia. Since important characters are not preserved in several specimens, eg. *Tarwinia* without preserved detailed structures of head and mouthparts [1,13], *P. tanlan* without mouthparts and with only partial mid- and hind legs, more Mesozoic materials are needed to fine tune the proposed phylogeny.

### Behavior of *Pseudopulex tanlan* sp. nov

The female *P. tanlan* is the first flea from the Mesozoic showing fully distended abdomen. All other known females of Mesozoic fleas are covered with dense setae on the abdomens, so it is difficult to identify the boundary between segments. Female *P. tanlan* is exceptional in having wide and clear segmental boundaries. Additionally, its ratio of the width of abdomen to the body length is about 0.43 vs. an average of 0.28 for other reported Mesozoic female fleas (Additional file 1: Table S1, see in [14]). Based on the ratio data above, we estimate the non-swelling abdominal width of the female *P. tanlan* is about 2.6 mm in contrast to 4.0 mm when fully distended. We consider that the most inflated 3^rd^ to 7^th^ segments of the distensible abdomen is a reservoir for holding its blood intake. Generally, examples of such broadly distended abdomens are very rare in the fossil record [21]. Due to the well-preservation of this fossil specimen without any distinct signs of disarticulation, we consider decomposition as less probable as the cause of abdominal swelling. However, we can not rule out the inflation caused by carrying eggs, even though the effect of egg load on abdominal size is much less significant as evidenced by extant haematophagous insects like mosquitoes, biting midges and others.

This is the first time that two Mesozoic fleas of *P. tanlan* with bodies in partially lateral view but heads in dorsal view (Figures 1A and 2A) are described. It is of interest to note that all Mesozoic fleas (except for *Tarwinia*) are preserved in dorsoventral position implying more or less dorsoventral depressed body in contrast to laterally compressed extant fleas and, by inference, *Tarwinia*. The female holotype of *P. tanlan* is preserved in partially lateral position, with all legs to one side, while the male paratype, in half-lateral position with legs directed to both sides. For the male fossil, its position can be interpreted as a result of a lateral direction of its robust legs which make it difficult for the insect taking a true lateral position, and a cylindrical to slightly compressed body somewhat turned sidewise during burial. In fact, this inference might be at least partly applicable to the male fossil of *Tarwinia* as well [13]. Unlike these, the fully distended body of female *P. tanlan* can be considered as near circular in section, and having comparatively weak legs, the insect was able to take almost any occasional position under the burial circumstances.

This inference that the female *P. tanlan* had a highly distensible abdomen which took nearly ovoid form under a complete blood load, make it possible for us to assess, even though roughly, the extent of its full diet. By calculating the volume changes from non-swelling to fully distended, we estimate an approximate volume of 0.02 cc (ml) of blood intake for *P. tanlan* (Additional file 2: Figure S1 see in [14]). This volume is 15 times of the volume that extant fleas are able to consume in a meal [22,23].

## Conclusions

Up to date, no more than 10 specimens of basal and transitional flea fossils have been collected from about 300 thousands of fossil insects in the CNU Lab. They have diverse and different morphological characters supporting the notion that the Cretaceous might be a significant transitional period for fleas, during which several groups with relatively reduced body sizes and other transitional characters gradually appeared (Figure 3). All these early fleas might have lived and fed on coexisting feathered dinosaurs, pterosaurs, primitive birds or medium-sized mammals coexisting in northeastern China [24-28]. These diverse hosts and their various life styles during the Early Cretaceous might be a driving force to enhance the flourishing and diversification of fleas.

### Ethics

The authors declare that the study makes no uses of human, clinical tools and procedures, vertebrate and regulated animal subjects and/or tissue, and plants.

## Availability of supporting data

The data set supporting the results of this article is available in the Dryad repository (https://datadryad.org), doi: 10.5061/dryad.q4jv0, see in [14].

## Additional files

Additional file 1: Table S1. Measurements for all the known female Mesozoic fleas. Additional file 2: Figure S1. Illustration of calculating the intake volume by holotype *Pseudopulex tanlan* sp. nov. (A and B) Selected parts of the fully distended abdomen; (C and D) Selected parts of the pseudomorph of non-swelling condition. (The additional files are available at https://datadryad.org, doi: 10.5061/dryad.q4jv0, see in [14]).
