# Supplementary material for: The first flea with fully distended abdomen from the Early Cretaceous of China
Source: BMC Evol Biol. 2014 Aug 27;14:168. doi: 10.1186/s12862-014-0168-1 (PMC4154525; doi:10.1186/s12862-014-0168-1)
Supplement: Additional file 1: Table S1. — Measurements for all the known female Mesozoic fleas. [file 12862_2014_168_MOESM1_ESM.doc]

Table S1. Measurements for all the known female Mesozoic fleas

| **Species** | **Body length (mm)** | **Width of abdomen at its widest point (mm)** | **Ratio (width of abdomen / body length)** | **Average of Ratio for specimens excluding**  ***Y. tanlan*** |
| --- | --- | --- | --- | --- |
| ***P. jurassicus*** | 17.00 | 4.0 | 0.24 | 0.28 |
| ***P. magnus*** | 22.82 | 6.1 | 0.28 |
| ***P. wangi*** | 14.80 | 5.1 | 0.34 |
| ***H. sinica*** | 20.00 | 6.0 | 0.30 |
| ***T. australis*** | 7.00 | 2.2 | 0.31 |
| ***S. longipes*** | 11.09 | 3.0 | 0.27 |
| ***S. exquisitus*** | 8.46 | 1.8 | 0.21 |
| ***P. tanlan* sp. nov.** | 9.26 | 4.0 | 0.43 |  |
