# Supplementary figures and images for: The first flea with fully distended abdomen from the Early Cretaceous of China
Source: BMC Evol Biol. 2014 Aug 27;14:168. doi: 10.1186/s12862-014-0168-1 (PMC4154525; doi:10.1186/s12862-014-0168-1)

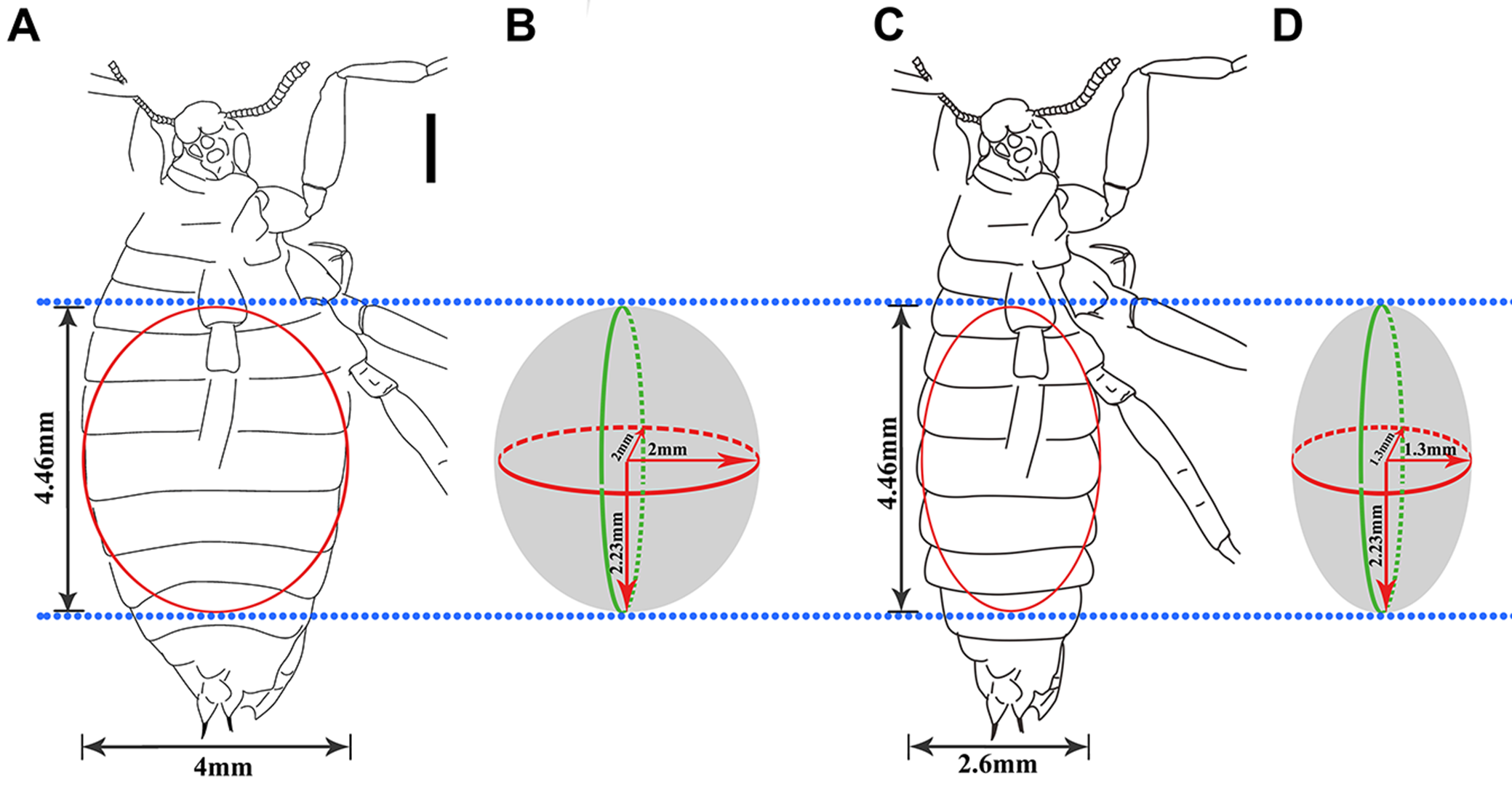

Supplement: Additional file 2: Figure S1. — Illustration of calculating the intake volume by holotype Pseudopulex tanlan sp. nov. (A and B) Selected parts of the fully distended abdomen; (C and D) Selected parts of the pseudomorph of non-swelling condition. (The additional files are available at https://datadryad.org, doi: 10.5061/dryad.q4jv0, see in [14]). [file 12862_2014_168_MOESM2_ESM.tiff]
